# Mycotoxins and Essential Oils—From a Meat Industry Hazard to a Possible Solution: A Brief Review

**DOI:** 10.3390/foods11223666

**Published:** 2022-11-16

**Authors:** Raluca Aniela Gheorghe-Irimia, Dana Tăpăloagă, Paul Rodian Tăpăloagă, Lucian Ionel Ilie, Cosmin Șonea, Andreea Iren Serban

**Affiliations:** 1 Faculty of Veterinary Medicine, University of Agronomic Sciences and Veterinary Medicine Bucharest, 105 Blvd, Splaiul Independenței, 050097 Bucharest, Romania; 2 Faculty of Animal Productions Engineering and Management, University of Agronomic Sciences and Veterinary Medicine Bucharest, 59 Blvd, Marasti, 011464 Bucharest, Romania; 3 Faculty of Biology, University of Bucharest, 91-95 Blvd, Splaiul Independenței, 050095 Bucharest, Romania

**Keywords:** meat, meat products, essential oils, mycotoxins, antifungal activity, food hazard, consumers’ health

## Abstract

The preservation of food supplies has been humankind’s priority since ancient times, and it is arguably more relevant today than ever before. Food sustainability and safety have been heavily prioritized by consumers, producers, and government entities alike. In this regard, filamentous fungi have always been a health hazard due to their contamination of the food substrate with mycotoxins. Additionally, mycotoxins are proven resilient to technological processing. This study aims to identify the main mycotoxins that may occur in the meat and meat products “Farm to Fork” chain, along with their effect on the consumers’ health, and also to identify effective methods of prevention through the use of essential oils (EO). At the same time, the antifungal and antimycotoxigenic potential of essential oils was considered in order to provide an overview of the subject. Targeting the main ways of meat products’ contamination, the use of essential oils with proven in vitro or in situ efficacy against certain fungal species can be an effective alternative if all the associated challenges are addressed (e.g., application methods, suitability for certain products, toxicity).

## 1. Introduction

Mycotoxins are considered an increasing health hazard, causing internal organ disorders, immunosuppression, or even death. Some mycotoxins, such as aflatoxins, ochratoxins, and fumonisins, have additional genotoxic effects and can be associated with certain types of cancer, as recognised by the International Agency for Research on Cancer (IARC) [1,2,3].

Current regulations on food safety and security are supposed to address all important aspects of the food pathogen ecology. Perhaps one of the most important issues at the moment is global climate change. In this respect, there are numerous studies that point to the fact that in the coming years there will be new combinations of mycotoxins, hosts, or geographical areas that will require distinct methods of approaching the diagnosis and studying toxigenic fungi. For example, Moretti et al. [4] made a documented prediction related to the extension of aflatoxin contamination risk in Central and Southern Europe over the next 30 years, through the *Aspergillus flavus* growth. The same situation is predicted for Northern, Central, and Southern Europe, regarding species of the genus *Fusarium*, and in Central and Northern Europe for *F. graminearum*.

The Food and Agriculture Organization of the United Nations (FAO) also estimates that about a quarter of the world’s total cereal production is currently contaminated with mycotoxins [5]. There are also documented cases where rivers may be contaminated with mycotoxins from crops grown with fungal-infected cereals, in addition to contamination via sewage systems [6].

Further alarm was given by the 2016 IARC and WHO (World Health Organization) press release on taking urgent action regarding the spread of mycotoxin contamination in developing countries [7].

Mycotoxin contamination of meat products has been documented as occurring through three routes: via spices and other contaminated raw materials (processing), via the presence of mycotoxin-producing mould on the surface of meat products from the environment, and the carry-over effect from animals exposed to contaminated feed [8].

Thus, human exposure may result indirectly from residual contamination of food of animal origin obtained from animals that have consumed contaminated feed. At the same time, the resulting residues may be of dual origin-the native toxin or metabolites that may partially or totally preserve the original toxic properties. In this direction, once ingested, mycotoxins are metabolised and are either eliminated, transformed into more toxic by-products, or accumulated. As a result, meat may be a vector for mycotoxins, resulting in sporadic cases of contamination [9].

On the other hand, in terms of finished product contamination, an example may be dry-cured meat products. In these foods, the mycoflora is complex, and it has been shown in vitro that some of the fungal species may be toxigenic. At the same time, the environment and processing conditions play an important role in mycotoxin synthesis [9].

In view of the above, risk management of mycotoxin development is mainly achieved by implementing preventive measures to control feed contamination, the processing environment, and the process itself (e.g., by controlling raw materials), as well as by setting legislative limits. Studies on the reducing and detoxifying properties of EO on mycotoxins, which are few in number, are mostly conducted in vitro, and further research is needed [9,10].

Globally, there are legislative regulations on animal products mainly for aflatoxins and, to a lesser extent, for ochratoxins. These depend on the data availability on toxicity and the carry-over effect. For example, in the European Union, regulations cover raw materials for animal consumption and milk, and there are particular situations in countries such as Serbia and Ukraine, where there are specific limits for meat and meat products. In most countries, legislative limits apply to all food for human consumption and not necessarily to meat or meat products [9].

Regarding the total daily intake data required by the main regulatory bodies, all the values are shown in Table 1.

**Figure 1:**
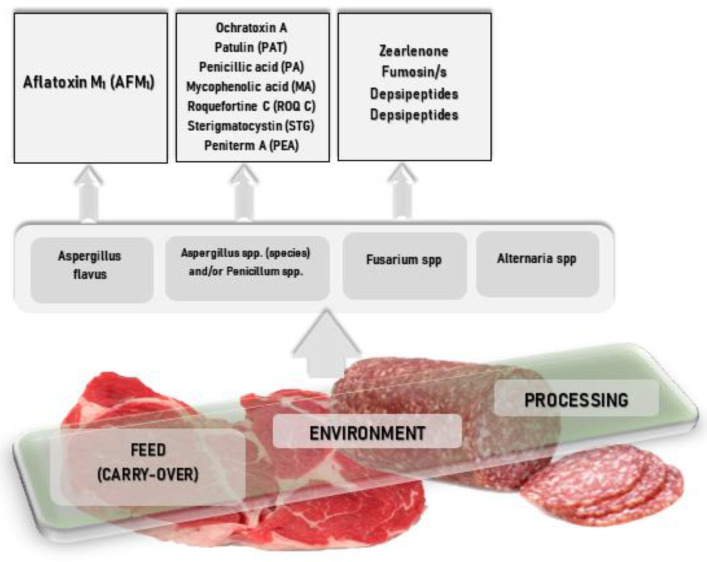
Main mycotoxins associated with animal products.

Most often, mycotoxin contamination is represented by metabolites of the *A. flavus* group (aflatoxin M1-milk and milk products, ochratoxin A-meat products, primarily from porcines), the others being present in lower proportions (Figure 1).

Fungal contamination is frequently obvious, as consumers are aware of the specific appearance and the dangers associated with consumption. On the other hand, when discussing the carry-over effect (contamination of spices or raw materials), the sensory changes in the finished product are not the most reliable indicator. Thus, with the new research in the field, tri-directional efforts (consumers, producers, and governmental institutions) should aim at identifying sustainable prevention alternatives, setting, and reviewing the mycotoxins limits, and raising consumer awareness [8].

In general, the prevention methods identification and possible decontamination of mycotoxins must take into account aspects such as the inactivation or irreversible destruction of mycotoxins and fungal spores and mycelia, the preservation of the organoleptic properties and nutritional value of foodstuffs, and cost-effectiveness [10].

In terms of possible treatment methods and prevention, the use of essential oils (EO) is an effective and forward-looking approach that can meet some of the above-mentioned criteria [10].

Currently, most of the attention is focused on the prevention of feed contamination, although there are areas globally where these measures are not sufficient, and the detoxification of feed substrates is also necessary. A study by Chilaka et al. [36] highlights the lack of government involvement in Africa, regarding the implementation of effective mycotoxin prevention measures and the major risks to food safety and security.

EO are considered GRAS (Generally Recognized as Safe) by the FDA (Food and Drug Administration), being active even in the vapour phase. Furthermore, in order to avoid sensorial changes in food products, modern technologies have developed encapsulation techniques, thus meeting the requirements multilaterally [37].

In addition to the growing consumer interest in buying healthier products, the issue of food pathogen resistance to synthetic preservatives is a common interest of food manufacturers. In this respect, there are studies indicating the antifungal and antimicrobial properties of EO, with minimal risk of resistance in terms of their chemical composition [38].

The aim of this paper is to identify the main mycotoxins associated with meat and meat products, their Total Daily Intake, and to document the essential oils that can be used along the “Farm to Fork” chain in order to inhibit fungal growth or to decontaminate the food substrate. However, given the reduced number of scientific studies on the antimycotoxigenic properties of EO, this article focuses mainly on the preventive potential of using EO for fungal inhibition, providing mentions on this issue where appropriate.

## 2. Mycotoxins Associated with Meat and Meat Products Contamination

The scientific studies investigating the natural occurrence of mycotoxins in meat products, and the observed concentrations are presented in Table 2. Subsequently, they have been characterised in terms of their associated health hazard, whilst identifying the EO with proven efficacy for each of them.

### 2.1. Ochratoxin A

Ochratoxin A (OTA) occurs naturally in food poisonings and is found in a variety of agricultural sectors. It is considered one of the most noxious mycotoxins, along with aflatoxin B_1_, and is commonly found in meat products [8]. It is produced by several fungi which require varying conditions (temperature and water activity), such as *Aspergillus carbonarius*, *Aspergillus niger*, *Aspergillus ochraceus*, *Penicillium verrucosum*, and others. In the meat industry, it is most often produced by *Aspergillus ochraceus* (8–37 °C, a_w_-0.95–0.99) and *P. verrucosum* (0–30 °C, a_w_-0.80) [65,66].

In terms of incidence, the route of meat product contamination is usually related to animal feed, with OTA being identified as residues [67]. This is due to the increased bioavailability and long half-life in certain monogastric animals such as pigs.

Some studies indicate that OTA absorption is predominantly gastrointestinal, with the distribution in the systemic circulation depending on the species: 40% chickens, 66% pigs, and 56% rabbits after oral administration. However, there is a variation in the absorption rate and attainment of maximum serum levels after single oral dosing (0.33 h chickens, 1 h rabbits, 10 h pigs) [68]. Other studies mention that the primary site for absorption is gastric, as the acidic environment enhances this phenomenon. In cattle, transport and accumulation of OTA is considered negligible due to the microbial metabolism in the rumen, but there are some discussions regarding animals with immature digestive systems [8].

Among food-producing animals, pigs seem to have the highest level of OTA, especially in Northern European countries. This is associated with the fat solubility of OTA [68].

In terms of maximum concentrations in specific tissues and organs, the highest were found in the kidney, lung, liver, blood, spleen, heart, and adipose tissue [66].

The analysis of the occurrence probability and risk severity indicates the involvement of OTA in the development of renal cancers in certain animal species, as well as the teratogenic, nephrotoxic, and immunosuppressive effects [65].

In regards to human health, a relatively conclusive association was made with a number of renal pathologies (Balkan Endemic Nephropathy and Chronic Interstitial Nephropathy), with OTA being classified by the IARC as a Group B2 carcinogenic [69]. For Balkan Endemic Nephropathy, the main features are associated with familial involvement, manifestations occurring after 15 years of living in an endemic area, as well as the presence of upper urothelial tract cancer. Chronic Interstitial Nephropathy has an acute or chronic evolution with manifestations occurring within days to months [65,70].

In animals, Perši et al. [71] observed that following an oral dosing of 300 μg/kg/day for 30 days in pigs, OTA accumulated in the kidneys, lungs, and adipose tissue, which resulted in minimal concentrations in final products such as black pudding frankfurters (14.02 μg/kg), liver sausage (13.77 μg/kg), and pâté (9.33 μg/kg).

However, given the increased incidence of OTA in food of animal origin, many countries have set stricter maximum limits and others have developed national guidelines. For example, in Romania, the maximum limit is 5 µg/kg in pig liver, kidney, and meat, while Italy developed national guidelines for achieving a limit of 1 µg/kg in pig meat and meat products [72].

In terms of reducing OTA levels in meat products, there are two general directions: prevention and decontamination. Prevention is achieved by following and implementing a food safety management system, while decontamination is achieved by using various physical and chemical treatments [73]. It is important to note that the use of chemical treatments (preservatives) may affect the sensory qualities of cured meat products and may contravene current consumer demands to purchase products without chemical residues, such as residues of fungicidal chemicals [74].

#### Applicable EO in Meat Products for the Prevention of OTA Development/Detoxification

As previously mentioned, perhaps the biggest challenge in terms of EO applicability is the changes in the sensory properties of the food products. Thus, in addition to the conventional methods, other practices to reduce the interference between EO and the food substrate have been identified using modern encapsulation technologies (nanoparticles, microencapsulation, active packaging).

Among the most effective EO are wild oregano (carvacrol, thymol), garlic, sage (camphor, borneol, 1,8-cineole), and peppermint (neomenthol, menthol, and menthone) [10].

Furthermore, in a study to demonstrate the efficacy of EO against OTA, Koteswara Rao et al. [75] concluded that neem and eucalyptus might also be efficient.

For cured products, Álvarez et al. [76] conducted a study on the efficacy of rosemary EO and *Debaryomyces hansenii* on artificially contaminated (*Penicillum nordicum*) dry-cured fermented sausages, during the ripening period. The application method varied by using rosemary as an ingredient for casings maceration or by the direct application of the EO, but the results were similar-decreased levels of OTA synthesis. Rosemary EO (alone or in combination with *D. hansenii*) reduced the number of proteins associated with OTA biosynthesis, affected cell wall integrity, and disrupted phenylalanine and ergosterol-associated proteins.

Regarding various food or beverage substrates, a study conducted on cocoa showed that *Aframomum danielli* EO demonstrated maximum OTA-reducing properties at a concentration of 2000 ppm [77]. Abd-El Fattah et al. [78] observed that using a concentration of 0.05% lemongrass EO in yoghurt resulted in a marked reduction of OTA and aflatoxins. Both EO have the potential to be the subject of further studies in meat products or feed.

### 2.2. Aflatoxins

Aflatoxins are specific to hot and humid areas and are produced by species of the genus *Aspergillus* (*A. flavus* and *A. parasiticus*). The most known and commonly found in food is aflatoxin B_1_, which has the highest carcinogenic and genotoxic potential of this group. Aflatoxin M_1_ is a metabolite of aflatoxin B_1_ and is often found in the milk of the animals fed contaminated feed [79].

Aflatoxins are characteristic of certain dried foods, spices, rice, corn, figs, cocoa beans, and others, and can contaminate before or after harvesting. In terms of incidence in meat products, OTA is most often identified, with AFB_1_ being detected less frequently and in lower concentrations [79]. In contrast, there has been multiple evidence of carry-over in tissues that can be found in the liver, muscle, and adipose tissue [8].

Aflatoxin B_1_ (AFB_1_) is classified by the IARC as a Group 1 human carcinogen and is associated with liver cancer [8].

In terms of the aflatoxin incidence in meat products, a study by Elzupir et al. [80] in Riyadh, KSA, showed that processed meat products had high levels of aflatoxins, with 37.5% of samples contaminated and a small percentage exceeding the permitted limits (4%).

Additionally, the cancer risk analysis from eating contaminated food identified a very high exposure limit. The most common types of aflatoxins were AFB_1_ and AFG_1_, concluding that this is of real importance for veterinary public health [80].

Furthermore, Shaltout et al. [81] observed from a study of one hundred samples that meatballs (kofta) had the highest level of aflatoxins.

In terms of contamination pathways, a study in Egypt found that the most frequent aflatoxin contamination of meat products was associated with the addition of spices [54].

#### Applicable EO in Meat Products for the Prevention of Aflatoxin Development/Detoxification

There are few studies on the actual application of EO to meat products, given their lower incidence. However, there are multiple studies on the efficacy of certain EO on the growth of *A. flavus* and *A. parasiticus*, some of which also have antiaflatoxigenic properties [82].

For example, a study by Masouri et al. [83] concluded that *Mentha piperita* EO could be used to suppress the effects of aflatoxins on various tissues. EO obtained from *Origanum vulgare* and *Ageratum conyzoides* were effective against aflatoxin B_1_ production in a study on maize and soybeans [84].

Razzaghi-Abyaneh et al. [85] reported a reduction of aflatoxin B_1_ up to 89.6% and up to 89.2% in aflatoxin G_1_ following the in vitro use of lime EO.

Neem EO caused irreversible inhibition of aflatoxin biosynthesis due to the alteration of the mycelial cell wall, according to a study by Abyaneh et al. [86].

Another EO with high potential for use in the meat processing and meat product industry is onion EO, with aflatoxin production inhibitory properties of 94.9% for *A. flavus* and 76.2% for *A. parasiticus* var. *globulosus* [87].

Other usable EO could be saffron, Zataria multiflora Boiss, Artemisia dracunculus, Callistemon lanceolatus, basil, Nigella sativa, coriander, dill seeds, and Boswellia sacra [88,89,90,91,92,93,94,95,96].

### 2.3. Zearalenone

Zearalenone (ZEA) is produced by many *Fusarium* species and is a nonsteroidal mycotoxin. Of its most known effects, the most important is the oestrogenic one, with synthetic derivatives of the mycotoxin being used in the meat processing industry as a growth promoter for cattle (Zeranol-α-ZAL). However, this practice is not accepted in the European Union. ZEA is also classified as a Group 3 carcinogenic risk by the IARC with the adverse health effects being a consequence of hormonal imbalance (various reproductive system disorders such as cervical, ovarian, and prostate cancer) [97,98].

Regarding the contamination of meat and meat products, Mirocha et al. [99] conducted a study on the distribution and determination of ZEA residues in broilers, concluding that the level of this mycotoxin was minimal under experimental conditions. In contrast, a 2014 study by Iqbal et al. [39] pointed out that 52% of poultry meat samples tested were contaminated with ZEA, with a maximum level of 5.10 µg/kg identified in the liver. Thus, the need for continuous monitoring of these mycotoxins in poultry meat was emphasized.

Another problem related to the meat processing industry and the presence of ZEA is associated with the contamination of beef and sheep meat. Some authors assume that the conversion of ZEA to zeranol takes place in the rumen and is an irreversible reaction. In this regard, numerous metabolic studies have determined increased levels of urinary excreted ZEA from cattle and sheep [100].

On the other hand, the identification of ZEA at the biliary and urinary levels led other authors to conclude that a distinction can be made between the natural presence of ZEA and that which is used to enhance industrial performance. This distinction is facilitated by the fact that α-zearalenol levels are always higher than those of zeranol—a factor of 5:1, making it possible to distinguish between abuse (<5:1) or contamination (>5:1) [100].

#### Applicable EO in Meat Products for the Prevention of ZEA Development/Detoxification

Parameters such as high humidity and low temperatures are known to promote zearalenone production by *Fusarium*. In terms of prevention, ZEA is quite stable at standard cooking temperatures, with the exception of high-pressure cooking and alkalinity. Thus, additional measures are needed [97,101].

For the purposes of this research, no studies on the reduction of ZEA levels by the addition of EO to contaminated meat products (decontamination and/or detoxification) were found, but there are numerous in vitro studies on the efficacy of the EO on ZEA production.

An example is the use of lemon, grapefruit, eucalyptus, and palmarosa EO, which have been shown to be effective, and even enhanced under controlled pH and temperature conditions [101]. On the other hand, another study by Velluti et al. [102] on maize, it was concluded that the efficacy of EO (oregano, cinnamon, lemongrass, clove, and palmarosa) is dependent on environmental conditions.

### 2.4. Citrinin

Citrinin (CIT-C_13_H_14_O_5_) is a polyketide-derived mycotoxin with hepatic and nephrotoxic effects, produced mainly by *Penicillium citrinum*, but also by other species of the genera *Penicillium*, *Aspergillus*, and *Monascus.* This mycotoxin was originally named monascidin A due to its identification in *Monascus* fermented products [19,22]. It is also noteworthy that it may be one of the most common contaminants globally, being produced mainly by the same fungi that produce OTA [23]. In this regard, the pathological health effects may even be additive, synergistic, or antagonistic to those produced by OTA.

The main organ of choice for CIT is the kidney, causing renal degeneration associated with weight loss, and is a possible causal agent of porcine nephropathy [19]. Although the main organ is the kidney, there are studies in which CIT has also been identified in the bone marrow. CIT is not classified as carcinogenic in humans and is classified as pertaining to Group 3 by the IARC [103].

In terms of the carry-over phenomenon, a study by Meerpoel et al. [104] on the effects of chronic citrinin consumption in pigs, broilers, and laying hens pointed out that there is a minimal contribution to increased CIT intake in humans, given the low rate of CIT transfer from feed to tissue for consumption.

An important problem is that of cured meat products. The surface of these traditionally obtained products is covered with species of the *Penicillium, Aspergillus*, and *Eurotium* genera. Studies have been conducted in which *Penicillium expansum* has been isolated from meat, producing CIT [23,105].

In another study conducted in Turkey by Sari et al. [106], it was observed that CIT was detectable at various concentrations (0.28–1.79 ng/g) in 36.84% of meat products samples and in one sausage sample (beef, lamb, and turkey). It was not detected in minced meat or salami samples.

Additionally, in another study certain fermented meat products were identified as co-contaminated with AFB_1_, OTA, and CIT [53].

In view of climate change, CIT levels have varied over the years. In this respect, it is important to consider the effect on the carry-over phenomenon. Meanwhile, the artisan-type meat products, obtained under uncontrolled conditions, might also be affected. In addition, depending on the integrity of the outer coating of meat products, mycotoxins may enter from the surface, similar to the OTA situation in dry-cured meat products during long-time ripening, and dry-fermented sausages during ripening [23].

For example, Wu et al. [107] observed considerable variations in citrinin levels in country-cured ham as being dependent on the storage temperature. At temperatures between 25–30 °C, citrinin concentrations were much higher compared to products stored at 15 °C, thus, temperatures lower than 15 °C are recommended for the storage and maturation of the susceptible products.

#### Applicable EO in Meat Products for the Prevention of CIT Development/Detoxification

Regarding the applicability of EO for the prevention of CIT production or detoxification in meat products, no specific studies were identified in the literature. On the other hand, there are studies on effective control strategies identification where EO of peppermint and neem leaf extract were mentioned as potential measures to inhibit CIT production. Experiments on in vitro inhibition of *P. citrinum* in cheese by means of *Z. multiflora Boiss* EO have also been performed [108].

Moreover, Aruna et al. [109] concluded that neem oil, eucalyptus, and olive EO could be effective in controlling *Aspergillus terreus* and CIT production.

### 2.5. Patulin

Patulin (PAT) is an uncomplex lactone produced by several species of *Penicillium* (most commonly *P. expansum*), *Aspergillus*, and *Byssochlamys*. This mycotoxin is most often identified in fruits and fruit products [110,111,112].

This mycotoxin is known for its potential toxigenicity to plant and animal tissues through reactions with the terminal sulfhydryl groups of proteins and polypeptides present in food [111].

Among the most known effects of PAT, immunological, gastrointestinal, and neurological effects are mentioned [111]. In studies on PAT toxicity using various experimental models, it was observed that the most common effects were cerebral and pulmonary oedema and haemorrhage, capillary destruction in the liver, and various types of nervous system damage, as well as multiple macrophage function inhibition [113]. PAT may also be of interest to veterinary public health due to its carcinogenic potential, being classified as a Group 3 by the IRAC [114].

In regards to the identification of PAT in meat products, it should be noted that it most often co-exists with other mycotoxins. Its toxicity depends largely on the pH of the food substrate being as acidic as possible, so PAT is quite stable [115]. In a study conducted by Bailly et al. [116], it was observed that PAT and OTA were not produced by toxigenic strains of *Penicillium* in dry-cured ham compared to CIT and cyclopiazonic acid. At the same time, after direct contamination, the initial PAT level decreased rapidly during the first hours of incubation at 20 °C.

Although limited, there are studies in which PAT has been identified in animal tissues intended for human consumption, an example being the results obtained by Cao et al. [41], where PAT was identified in pig liver.

#### Applicable EO in Meat Products for the Prevention of PAT Development/Detoxification

No studies were identified concerning the use of EO in food of animal origin for the prevention or detoxification of patulin. This is most likely due to the low incidence of this mycotoxin in animal tissues.

However, a study by Nguefack et al. [117] identified possible natural alternatives against *P. expansum* growth. In this study, *Ocimum gratissimum* EO was identified as effective, as well as the combination of *Cymbopogon citratus*, *Ocimum gratissimum*, and *Thymus vulgaris.*

Other effective EO could be tea tree, orange, and lemon [118,119].

### 2.6. Sterigmatocystin

Sterigmatocystin (STC) is a precursor of AFB_1,_ with a similar chemical structure, known to have significant carcinogenic effects [29,120]. In this regard, the CONTAM Panel of EFSA (European Food Safety Authority) conducted a comparative study for the carcinogenic potential of the two mycotoxins, concluding that the hazard associated with STC is approximately three orders of magnitude lower than AFB_1_ [29].

In terms of health effects, STC is classified by IRAC as pertaining to Group 2B [29]. The scientific opinion on the risk of STC to veterinary public health in the EFSA Journal states that although the carcinogenic risk is low, more data on exposure are needed.

Aflatoxins and STC cause similar toxic effects, predominantly affecting the kidneys and liver in acute toxicity cases [29].

Regarding the determination of STC in meat products, a study by Cao et al. [41] identified the presence of this mycotoxin in pork muscle in a concentration ranging from 0.76–1.23 μg/kg. Additionally, El-Kady et al. [121] observed in an experiment related to the mycotoxin production potential of fungi isolated from meat products that STC was produced by *E. Chevalieri*, *E. Chevalieri* var. *intermedium*, *E. amstelodami*, *E. pseudoglaucum*, and *E. rubrum*.

#### Applicable EO in Meat Products for the Prevention of STC Development/Detoxification

In terms of *A. versicolor* growth inhibition and STC production, onion (75%) and garlic (25%) EO were shown to have a synergistic effect [122]. Onion EO used individually completely inhibited STC production in *A. versicolor* at a concentration of 200 ppm [87]. In addition, Kocić-Tanackov et al. [123] demonstrated the efficacy of oregano EO against *Aspergillus* spp.

### 2.7. Fusarenon-X (4-acetylnivalenol)

Trichothecenes (TX) are mainly produced by *Fusarium* fungi and there are over 180 derivatives, which are divided into four types (A, B, C, and D) according to their functional groups. Fusarenon-X (FX) belongs to the B group of TX and is often associated with the contamination of feed and food for human consumption [5].

In terms of toxicity, it was observed in experimental studies that the maximum concentration of TX was found in the liver and kidney, concluding that these are the primary organs for the conversion of TX to NIV (Nivelanol—a secondary metabolite of TX). TX was also identified in the spleen in a study on piglets, 3 h after oral administration [5].

Organs primarily affected by TX are those with proliferative cells such as the spleen, thymus, testicles, small intestine, and hematopoietic tissues [5].

In terms of health effects, the chronic exposure of mice to TX and NIV most often results in reduced body mass, severe leukopenia, increased relative organ mass, and reduced feed efficiency. In addition, as there are not enough studies regarding carcinogenicity in humans, IARC has classified these toxins as belonging to Group 3. However, there are studies on various experimental models indicating tumour incidence caused by TX [124].

In addition, a study by Bony et al. [125] observed a genotoxic potential of FX and NIV, highlighting the need for more studies in this area.

Most often FX contaminates plant substrates such as wheat, barley, maize, and other cereals. There are also studies on the identification of FX in fish products such as gula substitutes. However, more studies are needed [51].

#### Applicable EO in Meat Products for the Prevention of FX Development/Detoxification

Trichothecenes, including FX, are found in relatively low concentrations in meat products, most often as a result of carry-over. In this regard, for the prevention of feed contamination, a study by Perczak et al. [126] concluded that *Cinnamomum zeylanicum*, *O. vulgare*, *Cymbopogon martini*, *Citrus aurantium dulcis*, *Thymus hiemalis*, *Mentha viridis*, *Foeniculum vulgare dulce*, and *Aniba rosaeodora* EO have a reduction effect on group B TX concentration levels of 94.51–100%.

### 2.8. T-2 Toxin

T-2 toxins are among the most relevant toxins in agriculture worldwide and are most often identified in grains. They belong to a family of chemically related toxins called trichothecenes, produced by species of the genera *Fusarium*, *Myrothecium*, and *Stachybotrys* [127].

The incidence of this mycotoxin is associated with developing countries and specific environmental conditions (substrate moisture, relative humidity, temperature, and oxygen availability) [127].

In terms of toxicity, T-2 toxins are some of the most toxic compared to the other members of the family to which it belongs. Poisoning in humans is known as Alimentary Toxic Allukemia (ATA) [127].

TX consumption can induce manifestations such as anorexia, emesis, carcinogenicity, haematotoxicity, neurotoxicity, and immunotoxicity [32]. Increased toxicity to mucous membranes and skin is also documented [128].

In terms of the risk of poisoning in humans, the consumption of contaminated animal products appears to be the primary cause [129].

In a study on carry-over for chickens, the EFSA-recommended limits for chicken feed of 0.25 mg/kg were found to be effective, resulting in minimal risk to human health [130].

Another study identified traces of T-2 toxins in back muscle, pig back fat, and chicken muscle in concentrations less than 0.5 μg/kg [68].

#### Applicable EO in Meat Products for the Prevention of T-2 Toxin Development/Detoxification

In order to prevent the carry-over effect, a study by Ancsin et al. [131] observed that the addition of garlic EO to broilers’ feed had desirable effects on some redox parameters.

### 2.9. Deoxynivelanol

Deoxynivelanol (DON) belongs to group B TX and is produced by *Fusarium graminearum* and *Fusarium culmorum*. The effects associated with this mycotoxin consumption include acute manifestations such as emesis, gastroenteritis, diarrhoea, and reduced food consumption with chronic implications [132].

DON exhibits increased thermostability under cooking and baking conditions, which is of interest in the food processing industry. It can also persist in meat after the consumption of contaminated feed or water by animals. According to the studies, this problem is associated with a minor risk, particularly when compared to the direct consumption of cereal products, but should be taken into account nonetheless [132].

Regarding the incidence in meat and meat products, a study by Zou et al. [43] identified DON as a residue in pig back fat samples at concentrations less than 0.5 μg/kg.

Furthermore, in an experimental administration of contaminated feed to pigs, traces of DON were detected in muscle (0.0016 ± 0.0016 mg/kg), liver (0.0057 ± 0.0043 mg/kg), and back fat (0.0002 ± 0.0004 mg/kg), which did not represent a hazard to the consumers [133].

#### Appliable EO in Meat Products for the Prevention of DON Development/Detoxification

According to a recent study, the effectiveness of lemon balm and palmarosa EO was observed, with better results obtained by controlling temperature and pH (20 °C, pH 3–6) [134].

Data on EO that may be used in the meat industry and their major components are summarised in Table 3.

## 3. Current Overview and Possible Solutions in Using EO in Meat and Meat Products Industry

As indicated, the use of EO in the meat industry, while promising in terms of efficiency, presents some notable challenges.

Firstly, one of the most important issues associated with the use of EO in meat and meat products is the toxic potential of some of them, even if they are considered GRAS by the FDA. Although there are many contradictory studies on this topic, it is necessary to carry out toxicity assessments prior to food product application, which can be difficult given the composition and active substances concentration heterogeneity. In this direction, various production and toxic or anti-nutritional substances disposal methods have been developed, and there are still ongoing studies. On the other hand, the EO Minimum Inhibitory Concentration (MIC) must also be considered, as very low doses are most often required to achieve an effect [135,136].

Secondly, the EO mechanism of action on fungi is quite controversial and, as in the mechanism of action on mycotxins’ structure, more studies are needed in this direction in order not to interfere with the meat and meat products’ sensory properties. So far there are several hypotheses, such as hydrogen bonds formed by the hydroxyl group, cellular respiration inhibition and loss of homeostasis by membrane modifications, acidification caused by phenolic compounds, mycotoxin and enzyme energy production systems blocking or membrane interactions (via the hydrophobic ring of benzene with aliphatic side chains or with membrane proteins) [10]. Regarding the EO mechanism against mycotoxins, no relevant studies could be found.

Thirdly, as mentioned above, the composition and concentration of the EO active substances may differ depending on the environmental conditions of the plant of origin and genetic diversity, which can make it difficult to use for both the carry-over prevention and use in the finished product (by incorporation, surface application, or packaging materials). Thus, various methods are needed to control and optimise parameters that may influence the standardisation processes of EO production [135,136].

Moreover, it is also important to address the toxicity of some EO to animals when used to counteract the carry-over effect. In this respect, there are studies suggesting their possible use as feed additives in certain concentrations [136,137,138], with other beneficial effects such as modulation of methane emissions by acting on methanogenic phenomena, antimicrobial resistance prevention, or as an alternative to legislative restrictions on the frequent use of zinc medications. There are also studies suggesting that the use of EO or other phytogenic feed additives is to be widely applied in the food animal industry in the context of restrictions on the use of antibiotics as growth promoters. In this respect, in the absence of scientifically endorsed recommendations regarding doses issued by the competent bodies, toxicological evaluation studies of EO prior to animal administration are necessary. It is important to underline, however, that as far as mycotoxins are concerned, the administration of EO to animals is not intended to reduce their tisular levels but to inhibit fungal growth in the ingested feed. Notably, in order to prevent the carry-over effect, there are also patented products based on EO. For example, patent RO131830 B1 is designed to limit the formation of aflatoxins and fumonisins in pre-harvest maize, TX in pre-harvest wheat, DON and ZEA in stored maize, and OTA in stored barley [139].

Additionally, by carrying out the digestion process, the concentration of active substances reaching the intestine is greatly reduced, even if there was a detoxifying potential of EO [135].

Next, apart from the low water solubility, volatility, and susceptibility to oxidation, they can affect the sensory qualities of meat products after application, especially when using oils with flavors that are not specific to certain meat products. For example, the study conducted by Sharma et al. [140], where different blends of EO were studied from a sensory point of view, concluded that, at increased concentrations, the level of acceptability of chicken sausage-type products is low. Bulai et al. [141] conducted a similar study on pork sausages made with lavender EO, obtaining the same results regarding overall acceptability.

From this point of view, techniques such as nanoencapsulation offer increased stability, bioavailability, and solubility with the use of a smaller amount of bioactive compound. Based on this technique, various nanostructures can be developed [142]. For example, Xavier et al. [143] used the nanoencapsulation technique to obtain a chitosan-based functionalized packaging with *Cinnamodendron dinisii*, attaining promising results on the preservation efficiency of minced beef.

A further possible solution regarding sensory properties may be to combine EO that have a potentiating effect on antifungal activity so that lower concentrations of those with an uncharacteristic flavor are used.

Another problem is the increased number of mycotoxins and the antifungal spectrum of EO. There are very few studies on the efficacy of EO on all types of mycotoxins affecting meat products. It should be noted, however, that some EO show an antifungal effect on several mycotoxigenic species. For example, oregano (*O. vulgare*) EO is effective against OTA, aflatoxins, zearalenone, sterigmatocystin, or fusarenon-X, cinnamon (*Cinnamomum zeylanicum*) EO is effective on zearalenone, ochratoxin A, Fusarenon-X and, eucalyptus EO on zearalenone, ochratoxin A, citrinin (Table 3).

Although some mycotoxins have a higher incidence rate than others, issues such as climate change could modify this parameter [144].

Another issue that needs to be addressed is the insufficient research on the detoxifying or antimycotoxigenic potential of essential oils, a topic that could have real promise. In this respect, most of the EO that could be used in the meat and meat products industry are aimed at preventing the growth of mycotoxigenic fungi Undeniably, however, there is a great need for effective prevention measures [145,146].

Meanwhile, regarding the economical aspects, although some studies claim that the use of EO can be a low-cost method of preventing mycotoxigenic fungi, there are some associated problems [136,147]. The cost of obtaining essential oils is also influenced by the amount of EO in the original plant and the extraction method [147]. Mainly, plant extracts must be subjected to investigations prior to approval, patent, and market. From this perspective, all the procedures that involve the direct contact of EO with food must be patented, along with toxicity studies. All the above-mentioned involve high additional costs depending on the country (e.g., income, legislation, flora, traditions) and a relatively difficult process for the operators, in terms of time, technology, and finances [148].

Furthermore, the application method is very important from an economical point of view. For example, regarding the antibacterial effect, it is known that the efficacy of EO is increased when they come into direct contact with microorganisms, and thus with meat or meat products, this method is costly as very large quantities of EO are required [149]. In this regard, no studies were found on the EO application methods’ efficiency in the case of antifungal growth or antimycotoxigenic purposes. On the other hand, other EO application methods, such as active packaging systems are incompatible with European Union legislation [150].

## 4. Conclusions

The use of EO in meat and meat products as antifungal or antimycotoxigenic substances is subordinated to the level of subject knowledge. The future prospects are based on achieving the desideratum of sustainability, safety, and cost, scientist intervention to overcome the associated challenges being essential.

First, the toxic potential of these plant extracts must be considered, depending on the stage at which they are used along the “Farm to Fork” chain. In the absence of legislative recommendations on dosage, toxicological studies should be carried out prior to the addition of EO to meat and meat products or feed.

The diversity of the active compounds in EO according to the plant of origin should also be addressed. EO stabilisation and standardisation methods are also recommended. Subsequently, considering that EO are also flavouring agents, tests on the consumers’ or animals’ acceptability are required.

EO food matrix application research is mandatory in order to clarify the exact mechanisms of action on fungi or mycotoxins for prevention or decontamination.

Even if EO direct application in meat and meat products is subjected to problems such as changes in sensory parameters and difficulty of application due to the possibility of oxidation, lack of solubility in water, or volatility, there are numerous studies that have identified some alternatives or solutions to this problem, such as nanoencapsulation or active packaging.

There are rather limited resources regarding the identification of EO with organoleptically acceptable aromas, along with a very broad spectrum of toxigenic fungi producing mycotoxins identified in meat products. Possible combinations between these plant extracts require further studying.

Future research may involve the study of EO that have had specific antifungal or antimycotoxigenic efficacy in plant substrates or milk products and extend them in meat and meat products.

Additionally, measures are demanded to ease the EO testing, patenting, and marketing procedures in terms of time and costs along with identifying cost-effective methods of applying them in line with legislative regulations and natural product consumption trends.

In conclusion, given the incidence of mycotoxins in meat and meat products and the prospects for its increase, the use of EO as a replacement for the classical antifungal agents and physical and chemical mitigation treatments may be a sustainable solution.

## Figures and Tables

**Table 1:** Total daily intake for mycotoxins and median lethal toxicity.

Mycotoxin	Total Daily Intake (TDI)		Toxicity LD_50_ (mg/kg bw)	Health Effects	References
Ochratoxin A	European Food Safety Authority	120 ng/kg bw/week	20–25 mg/kg^−1^ in humans	Nephrotoxicity hepatotoxicity, immunotoxicity, neurotoxicity, teratogenicity, and carcinogenicity	[11,12]
	Joint FAO/WHO Expert Committee on Food Additives (JECFA)	100 ng/kg bw/week			
	Scientific Committee of Food (SCF) of the European Union	5 ng/kg bw/day			
Aflatoxin B_1_	European Food Safety Authority	4 µg/kg to 10 µg/kg for total aflatoxins	0.36 mg/kg body weight in humans	Genotoxicity, hepatotoxicity, immunotoxicity, teratogenicity, carcinogenicity	[13,14,15,16]
	Joint FAO/WHO Expert Committee on Food Additives (JECFA)	Not more than 10 µg/kg for total aflatoxin of which aflatoxin B_1_ shall not be more than 5 µg/kg			
	Scientific Committee of Food (SCF) of the European Union	5–10 µg/kg for total aflatoxins			
Aflatoxin B_2_	European Food Safety Authority	4 µg/kg to 10 µg/kg for total aflatoxins	1.7 mg/kg bw in duck (oral)	Hepatotoxicity, carcinogenicity, weak mutagenic effects	[13,14,15,17]
	Joint FAO/WHO Expert Committee on Food Additives (JECFA)	Not more than 10 µg/kg for total aflatoxin of which aflatoxin B_1_ shall not be more than 5 µg/kg			
	Scientific Committee of Food (SCF) of the European Union	5–10 µg/kg for total aflatoxins			
Aflatoxin G_2_	European Food Safety Authority	4 µg/kg to 10 µg/kg for total aflatoxins	2.5 mg/kg in ducklings (oral) NA	Low toxicity	[13,14,15,17,18]
	Joint FAO/WHO Expert Committee on Food Additives (JECFA)	Not more than 10 µg/kg for total aflatoxin of which aflatoxin B_1_ shall not be more than 5 µg/kg			
	Scientific Committee of Food (SCF) of the European Union	5–10 µg/kg for total aflatoxins			
Zearlenone	European Food Safety Authority	0.25 µg/kg body weight	Between 2000 and 20,000 mg/kg^−1^ in rodents and guinea pigs	Reproductive toxicity, hepatotoxicity, immunotoxicity, genotoxicity and carcinogenicity, intestinal toxicity, endocrine disruption	[19,20,21]
	Joint FAO/WHO Expert Committee on Food Additives (JECFA)	0.5 µg/kg bw			
Citrinin	European Food Safety Authority	0.2 µg/kg b.w. per day	35–58 mg/kg^−1^ in an oral administration to a mouse, 50 mg/kg^−1^ to a rat, 57 mg/kg^−1^ to a duck, 95 mg/kg^−1^ to a chicken, and 134 mg/kg^−1^ to a rabbit	Necrotic changes of parenchyma organs ephrotoxicity, gastrointestinal ailments, fetal malformations, and lymphoid tissue damage (additively, synergistically, or antagonistically to OTA)	[22,23]
Patulin	Joint FAO/WHO Expert Committee on Food Additives (JECFA)	0.4 µg/kg bw	5 mg/kg in mice (IP)	Lung congestion, epithelial cell degeneration, along with carcinogenic, genotoxic, immunosuppressive, and teratogenic effects	[24,25,26]
	Scientific Committee of Food (SCF) of the European Union	0.4 ug/kg bw			
Sterigmatocystin	European Food Safety Authority	Not established due to the lack of data	32 mg/kg bw for sterigmatocystin dissolved in dimethylsulfoxide (DMSO) in Vervet monkeys 800 mg/kg in mice (oral)	Possible carcinogen, immunotoxic and immunomodulatory activity, together with mutagenic effects	[27,28,29]
	Joint FAO/WHO Expert Committee on Food Additives (JECFA)	Not established			
	Scientific Committee of Food (SCF) of the European Union	–			
Fusarenon-X (4-Acetylnivalenol)	European Food Safety Authority	Not established	3.3 mg/kg in mice (i.p.)	Immunosuppression, intestinal malabsorption, developmental toxicity, and genotoxicity	[5,30,31]
	Joint FAO/WHO Expert Committee on Food Additives (JECFA)	Not established			
	Scientific Committee of Food (SCF) of the European Union	Not established			
T-2 Toxin	European Food Safety Authority	100 ng/kg b.w. for T-2 toxins and HT-2 toxins	2–4 mg/kg^−1^ in mice	Anorexia, emesis, carcinogenicity, haematotoxicity, neurotoxicity and immunotoxicity	[11,32,33,34,35]
	Joint FAO/WHO Expert Committee on Food Additives (JECFA)	25 ng/kg bw for T-2, HT-2 and DAS, alone or in combination			
	Scientific Committee of Food (SCF) of the European Union	0.06 g/kg bw/day. for T-2 toxins and HT-2 toxins			

**Table 2:** Mycotoxins identified in meat and meat products.

Type	Food Product	Mycotoxin	Concentration	References
MEAT	Chicken meat	Aflatoxin/S	≤8.01 μg/kg	[39]
		Ochratoxin A	0.38 μg/kg	[40]
		Zearalenone	≤5.10 μg/kg	[39]
	Pig muscle	Aflatoxin B_1_	0.46–0.74 μg/kg	[41]
		Sterigmatocystin	0.76–1.23 μg/kg	[41]
		Ochratoxin A	≤0.04–0.06 μg/kg	[42]
		T-2 Toxin	0.0240–0.4515 μg/kg	[43]
	Pork meat	Ochratoxin A	≤0.14 μg/kg	[44]
		Zearalenone	≤4.31 μg/kg	[45]
	Duck meat	Ochratoxin A	0.09 μg/kg	[46]
	Fish	Aflatoxin B1	tr-moderately high	[47]
		Aflatoxin B_2_	1.2 μg/kg	[48]
		Aflatoxin G_1_	tr-moderately high	[47]
		Aflatoxin G_2_	tr-moderately high	[47]
		Aflatoxin/S	>9.9–20.4 μg/kg	[49]
		Ochratoxin A	0.5–1.4 μg/kg	[48]
		Enniatin A_1_	1.7–6.9 μg/kg	[50]
		Enniatin B	7.0 μg/kg	[51]
		Enniatin B_1_	1.4–31.5 μg/kg	[50]
		Fusarenon-X (4-Acetylnivalenol)	4.0 μg/kg	[51]
		Zearalenone	11.2–14.8 μg/kg	[48]
MEAT PRODUCTS	Fish products	Aflatoxin/S	3.8 μg/kg	[52]
	Meat products (Dry-meat products)	Aflatoxin B_1_	<LOQ-3.0 μg/kg	[53]
		Aflatoxin/S	1.0 μg/kg	[52]
		Citrinin	<LOQ-1.3 μg/kg	[53]
		Ochratoxin A	<LOQ- ≤ 7.83 μg/kg	[53]
	Hot dog	Aflatoxin B_1_	5 μg/kg	[54]
		Aflatoxin B_2_	2 μg/kg	[54]
		Ochratoxin A	0.38 μg/kg	[44]
	Ham	Aflatoxin B_1_	0.95–1.06 μg/kg	[55]
		Ochratoxin A	≤28.42 μg/kg	[42]
	Salami	Ochratoxin A	≤0.08 μg/kg	[42]
	Sausage	Aflatoxin B_1_	1.5 μg/kg	[53]
		Aflatoxin B_2_	3 μg/kg	[54]
		Citrinin	1.0 μg/kg	[53]
		Ochratoxin A	0.12 μg/kg	[44]
		Zearalenone	2.1–8.9 μg/kg	[56]
ORGAN MEATS	Cow liver	Ochratoxin A	14 μg/kg	[57]
	Pig liver	Aflatoxin B_1_	0.2–0.87 μg/kg	[58]
		Aflatoxin B_2_	0.52 μg/kg	[41]
		Aflatoxin M_1_	0.20–0.44 μg/kg	[59]
		Citrinin	1.45 μg/kg	[41]
		Ochratoxin A	≤0.61 μg/kg	[60]
		Patulin	0.69 μg/kg	[41]
	Chicken liver	Aflatoxin B_1_	0.61–2.48 μg/kg	[61]
		Aflatoxin/S	0.02–0.049 μg/kg	[62]
		Citrinin	0.89 μg/kg	[41]
		Ochratoxin A	0.14–3.90 μg/kg	[63]
		Zearalenone	40.0–74.0 μg/kg	[64]
	Chicken heart	Zearalenone	49.3–87.5 μg/kg	[64]
	Chicken gizzard	Aflatoxin B_1_	0.81–1.34 μg/kg	[61]
		Ochratoxin A	0.25–9.94 μg/kg	[63]
		Zearalenone	39.9–84.9 μg/kg	[64]

**Table 3:** EO used in experimental studies for mycotoxins prevention or decontamination.

Mycotoxin	EO (Antifungal/Antimycotoxigenic)	Major Constituents/Main Fungicidal Substances	References
Ochratoxin A	*Cinnamomum zeylanicum*	Cinnamaldehyde, citral, eugenol	[10,75,76,77,78]
	*Origanum vulgare*	Carvacrol, thymol	
	*Allium sativum*	Allicin, alliin, diallyl sulfide, diallyl disulfide, diallyl trisulfide, ajoene, and S-allyl-cysteine	
	*Salvia officinalis*	Camphor, borneol, 1,8-cineole	
	*Azadirachta indica*	Azadirachtin, nimbolinin, nimbidin, nimbidol, sodium nimbinate, gedunin, salannin, and quercetin	
	*Eucalyptus*	1,8-cineole, α-pinene, α-phellandrene, and p-cymene	
	*Rosmarinus officinalis*	1,8-cineole, camphor, α-pinene, limonene, camphene and linalool	
	*Cymbopogon citratus*	Geranial, neral, myrcene	
	*Aframomum danielli*	1,8-cineole, β-pinene, α-terpineol, α-pinene, and α-terpinyl acetate	
	*Mentha*	Neomenthol, menthol and menthone	
Aflatoxin B_1_ Aflatoxin B_2_ Aflatoxin G_2_	*Ocimum basilicum*	Methyl eugenol, methyl chavicol	[82,87,88,89,90,91,92,93,94,95,96]
	*Thymus vulgaris*	p-cymene, γ-terpinene, thymol	
	*Mentha viridis*	Neomenthol, menthol and menthone	
	*Mentha piperita*	Neomenthol, menthol and menthone	
	*Origanum vulgare*	Carvacrol, thymol	
	*Minthostachys verticillata*	Pulegone, menthone, limonene	
	*Matricaria chamomilla*	α-bisabolol oxide	
	*Calendula officinalis*	τ-muurolol, β-eudesmol, α-cadinol, δ-cadinene	
	*Achillea millefolium*	β-pinene, sabinene, 1,8-cineole, β-caryophyllene, (E)-nerolidol, guaiol, chamazulene	
	*Achillea fragrantissima*	Santolina alcohol, artemisia alcohol, artemisia ketone, cis-thujone, trans-thujone	
	*Pimpinella anisum*	Trans-anethole	
	*Carum carvi*	Carvone, limonene, b-myrcene	
	*Foeniculum vulgare*	Trans-Anethole, alpha-pinene, limonene	
	*Cinnamomum zeylanicum*	Cinnamaldehyde, citral, eugenol	
	*Agrimonia eupatoria*	Cedrol, α-pinene, linalool, α-terpineol, bornyl acetate, eucalyptol	
	*Peumus boldus*	Ascaridol, 1,8-cineole, terpineol, terpinene-4-ol, γ-terpinene, safrole	
	*Crocus sativus*	Safranal, picrocrocin, crocin	
	*Zataria multiflora Boiss*	Carvacrol, terpinene, pinene	
	*Artemisia dracunculus*	Stragole	
	*Callistemon lanceolatus*	1,8-cineole, -pinene	
	*Nigella sativa*	TQ, ρ-cymene, carvacrol, t-anethole, 4-terpineol, longifolene	
	*Coriandrum sativum*	Linalool	
	*Anethum graveolens* L.	α-phellandrene, dill ether, limonen	
	*Boswellia sacra*	duva-3,9,13-trien-1,5α-diol-1-acetate, octyl acetate	
	*Citrus aurantiifolia*	Limonene, linalool, citronellal, citronellol	
Zearalenone	*Citrus aurantiifolia*	Limonene, linalool, citronellal, citronellol	[101,102]
	*Eucalyptus*	1,8-cineole, α-pinene, α-phellandrene, and p-cymene	
	*Citrus paradisi*	D-Limonene	
	*Cymbopogon martinii*	Geraniol, geranyl acetate, linalool	
	*Origanum vulgare*	Carvacrol, thymol	
	*Cinnamomum zeylanicum*	Cinnamaldehyde, citral, eugenol	
	*Syzygium aromaticum*	Eugenol, β-caryophyllene, eugenyl acetate	
	*Cymbopogon citratus*	Geranial, neral, myrcene	
Citrinin	*Zataria multiflora Boiss*	Carvacrol, terpinene, pinene	[108,109]
	*Azadirachta indica*	Azadirachtin, nimbolinin, nimbidin, nimbidol, sodium nimbinate, gedunin, salannin, and quercetin	
	*Eucalyptus*	1,8-cineole, α-pinene, α-phellandrene, and p-cymene	
Patulin	*Ocimum gratissimum*	Eugenol	[117,118,119]
	*Cymbopogon citratus* *Ocimum gratissimum* *Thymus vulgaris*	Geranial, neral, myrcene p-cymene, γ-terpinene, thymol, eugenol	
Sterigmatocystin	*Allium cepa*	Dipropyl disulfide, dipropyl trisulfide	[122,123]
	*Origanum vulgare*	Carvacrol, thymol	
	*Allium sativum*	Allicin, alliin, diallyl sulfide, diallyl disulfide, diallyl trisulfide, ajoene, and S-allyl-cysteine	
Fusarenon-X (4-Acetylnivalenol)	*Cinnamomum zeylanicum*	Cinnamaldehyde, citral, eugenol	[126]
	*Origanum vulgare*	Carvacrol, thymol	
	*Cymbopogon martinii*	Geraniol, geranyl acetate, linalool	
	*Citrus aurantium dulcis*	D-limonene	
	*Thymus hyemalis*	Thymol, p-cymene, γ-terpinene	
	*Mentha viridis*	Neomenthol, menthol and menthone	
	*Foeniculum vulgare*	Trans-Anethole, alpha-pinene, limonene	
	*Aniba rosaeodora*	Linalool	
T-2 Toxin	*Allium sativum*	Allicin, alliin, diallyl sulfide, diallyl disulfide, diallyl trisulfide, ajoene, and S-allyl-cysteine	[131]
Deoxynivelanol	*Citrus aurantiifolia*	Limonene, linalool, citronellal, citronellol	[134]
	*Cymbopogon martinii*	Geraniol, geranyl acetate, linalool	
